# An at‐home laboratory in plant biology designed to engage students in the process of science

**DOI:** 10.1002/ece3.8441

**Published:** 2021-12-15

**Authors:** Laura J. Schnell, Gavin L. Simpson, Danae M. Suchan, William Quere, Harold G. Weger, Maria C. Davis

**Affiliations:** ^1^ Department of Biology University of Regina Regina Saskatchewan Canada; ^2^ Institute for Microbial Systems and Society University of Regina Regina Saskatchewan Canada; ^3^ Department of Animal Science Aarhus University Tjele Denmark

**Keywords:** at‐home experiment, course‐based research, remote learning, undergraduate research experience

## Abstract

The COVID‐19 pandemic prompted a transition to remote delivery of courses that lack immersive hands‐on research experiences for undergraduate science students, resulting in a scientific research skills gap. In this report, we present an option for an inclusive and authentic, hands‐on research experience that all students can perform off‐campus. Biology students in a semester‐long (13 weeks) sophomore plant physiology course participated in an at‐home laboratory designed to study the impacts of nitrogen addition on growth rates and root nodulation by wild nitrogen‐fixing Rhizobia in *Pisum sativum* (Pea) plants. This undergraduate research experience, piloted in the fall semester of 2020 in a class with 90 students, was created to help participants learn and practice scientific research skills during the COVID‐19 pandemic. Specifically, the learning outcomes associated with this at‐home research experience were: (1) generate a testable hypothesis, (2) design an experiment to test the hypothesis, (3) explain the importance of biological replication, (4) perform meaningful statistical analyses using R, and (5) compose a research paper to effectively communicate findings to a general biology audience. Students were provided with an at‐home laboratory kit containing the required materials and reagents, which were chosen to be accessible and affordable in case students were unable to access our laboratory kit. Students were guided through all aspects of research, including hypothesis generation, data collection, and data analysis, with video tutorials and live virtual sessions. This at‐home laboratory provided students an opportunity to practice hands‐on research with the flexibility to collect and analyze their own data in a remote setting during the COVID‐19 pandemic. This, or similar laboratories, could also be used as part of distance learning biology courses.

## 1 INTRODUCTION

Undergraduate laboratories, whether standalone courses or as part of a traditional laboratory plus lecture course format, are an essential part of the undergraduate Science, Technology, Engineering, and Mathematics (STEM) curriculum that help students practically apply the theoretical concepts introduced in lectures. Laboratory activities are used as a pedagogical tool for practicing real‐world problem‐solving and critical thinking and often result in greater intellectual curiosity and appreciation for the natural world (Holt et al., 1969). In undergraduate science programs, laboratory courses offer students hands‐on experience with relevant techniques, specimens, and scientific equipment. These hands‐on learning experiences in laboratory courses engage multiple senses (sight, touch, and smell) during the act of the investigation and are generally associated with improved learning outcomes (Gya & Bjune, 2021; Willis, 2007). However, not all laboratory activities provide equal benefit to students. Some laboratory activities, such as those that follow a short, highly structured format that leads to a known answer (colloquially called “cookbook” laboratories), may fail to allow students to navigate uncertainty, deal with errors, or challenge misconceptions (Davis et al., 2020; Shortlidge & Brownell, 2016).

In contrast, authentic research‐based laboratory courses, such as course‐based undergraduate research experiences, and inquiry‐based laboratory courses, almost always benefit students, including the least‐prepared. For example, Blumer and Beck (2019) recently reported that inquiry‐based laboratory courses implementing authentic research experiences improved the scientific reasoning of students scoring in the lowest quartile following a pretest. The growing body of evidence demonstrates that courses offering authentic research experiences result in increased content knowledge, with students reporting higher self‐confidence in performing scientific laboratory tasks and thinking like a scientist, ultimately leading to increased student persistence in STEM fields (Beck & Blumer, 2012; Cooper et al., 2019, 2020; Cooper & Brownell, 2018; Cooper, Gin, et al., 2019; Corwin et al., 2018; Graham et al., 2013; Hark et al., 2011; Harrison et al., 2011; Rodenbusch et al., 2016; Sanders & Hirsch, 2014; Ward et al., 2014).

The COVID‐19 pandemic has necessitated innovation in teaching, especially regarding practical laboratory courses in life sciences (Davis et al., 2020). Many educators teaching life sciences implemented virtual laboratory experiences where possible, in fields such as biochemistry (Guarracino, 2020; White et al., 2002), ecology (Hines et al., 2020; Wu et al., 2016), medical sciences (Doubleday et al., 2011; Holmberg et al., 2021; Moreno‐Ger et al., 2010; Šorgo et al., 2008), and microbiology (Dustman et al., 2021; Makransky et al., 2019). The abrupt shift to online and remote learning in the past year prompted a wide range of teaching and learning strategies for hands‐on scientific skills. Some educators hosted “choose your adventure” remote experiments, where students designed experiments and directed the instructor or teaching assistant (in live sessions via video conferencing software) to perform tasks in the laboratory. Other educators implemented virtual laboratory simulation software such as Labster (www.labster.com) and Interactive Laboratory Microbiology (www.interactivelabmicro.com); however, these can be cost‐prohibitive in the long term due to subscription prices and restricted budgets in higher education. Other educators designed data‐focused laboratories, where instructors or teaching assistants collected the relevant data for students to perform guided data analysis (Papaneophytou, 2020). At‐home interactive laboratories, which involve physically manipulating the study system or organism at a location other than the campus laboratory, have also been reported (Andrews et al., 2020; Creech & Shriner, 2020; Fox et al., 2020; Gya & Bjune, 2021).

Providing students with the opportunity to participate in equitable, inclusive, and authentic research‐based laboratory experiences is essential to successfully prepare undergraduates for advanced courses, graduate school, and prospective STEM careers. This has become especially important during the COVID‐19 pandemic, with the identification of a skill gap that is developing within the current student cohort due to the lack of hands‐on laboratory experience that is critical across many STEM disciplines (Baker & Cavinato, 2020; Noel et al., 2020). At‐home laboratories can be used to bridge this skills gap through increased learning flexibility as they allow students to gain practical and applied skills in a remote (i.e., off‐campus) setting. However, considering the socio‐economic and geographic diversity in our student population, at‐home laboratory activities must be designed with careful consideration for equity and inclusion (Creech & Shriner, 2020; Fox et al., 2020; Gya & Bjune, 2021). There are currently few examples of innovative and equitable open‐ended research experiences in biology that do not require expensive supplies and equipment and that can be safely performed at home (Creech & Shriner, 2020; Gya & Bjune, 2021).

Here, we describe an open‐ended research experiment for a 200‐level (Sophomore) Plant Physiology laboratory course that is designed to be conducted at home over the course of a semester. This authentic, at‐home undergraduate research experience (URE) was designed with the goal of giving students an opportunity to apply scientific processes they will use throughout their undergraduate, graduate, and work careers (form a hypothesis, design an experiment, collect and analyze data) and to guide students through quantitative reasoning, as recommended in Vision and Change in Undergraduate Biology Education: A Call to Action (American Association for the Advancement of Science (AAAS), 2011).

The experiment and subsequent data analysis is performed in two parts: (1) students study the effect of nitrogen fertilizer on *Pisum sativum* (Pea) growth rate and root nodulation by wild nitrogen‐fixing Rhizobia using local soils independently sourced by students, and (2) the collected data are pooled and shared among the students so that they can perform guided data analysis using the open‐source statistical software R (R Core Team, 2021). The concepts in this laboratory directly apply the content covered in the accompanying Plant Physiology course lectures and encourage students to consider the practical applications of nutrient cycling and how it relates to agricultural practices. Students were provided with a laboratory kit to perform this experiment at home; however, the experiment was designed such that the required materials can be easily sourced by international students.

### 1.1 Analyzing the impact of nitrogen on pea plant growth and root nodulation

#### 1.1.1 The at‐home laboratory kit

**FIGURE 1:**
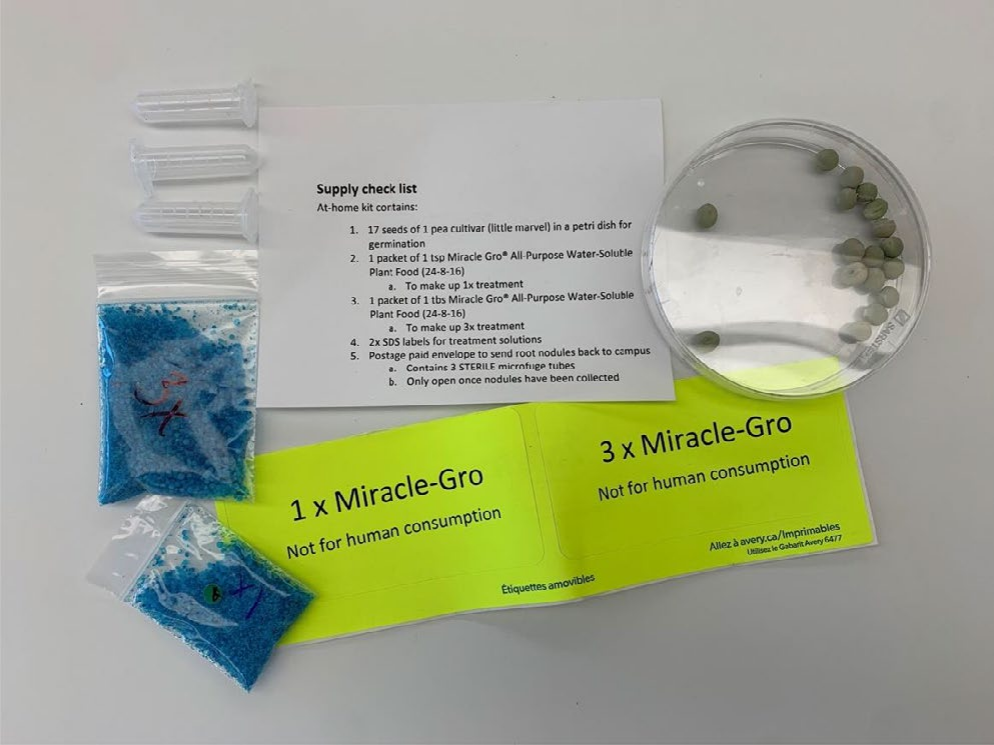
Contents of the at‐home laboratory kit provided to students in Fall 2020. Postage paid envelope for mailing nodules harvested following the at‐home experiment is not pictured. Photo: Laura J. Schnell

The at‐home laboratory kit (Figure 1) contained 21 pea seeds (Little Marvel cultivar) and one 100‐mm petri dish for germination. Premeasured Miracle Gro^®^ All‐Purpose Water‐Soluble Plant Food (“24‐8‐16,” equivalent to 24% nitrogen, 3.5% phosphorus, and 13.3% potassium by mass) was provided for two treatment levels (1X and 3X) with preparation instructions detailing how to bring the treatment solutions to the final concentrations using tap water. Students were instructed to prepare a “control” of 0X Miracle Gro^®^ containing only tap water. The kit also contained fluorescent colored Safety Data Sheet sticker labels that the students were instructed to apply to solution storage containers, and instructions for safely storing containers out of reach of children and pets. Additional materials that must be provided by the student include the following: three sealable treatment solution containers, plant pots, and local soil. The kit was mailed out to students who were unable to pick up the kit (Canada only). As previously stated, materials in the at‐home kit were chosen to be affordable and easily sourced if students were unable to access our kit. International students were provided with the list of materials to be sourced. All students were provided with the option to perform an alternative, virtual only experiment if they were unable to perform the experiment for any reason.

#### 1.1.2 The nitrogen addition experiment—background

This experiment examines nodulation in pea (*P*. *sativum* L.) roots. Pea plants are members the Fabaceae/Leguminosae (the legume family); there are approximately 19,500 legume species, making it the third largest plant family (Christenhusz & Byng, 2016). Most legume species, including peas, are capable of biological nitrogen fixation (BNF) via a symbiotic association with soil bacteria known as rhizobia. BNF is catalyzed by the prokaryotic (certain bacteria and archaea) enzyme nitrogenase, which catalyzes the ATP‐dependent “fixation” of atmospheric N_2_ into ammonia (NH_3_, which is in equilibrium with ammonium, ${\text{NH}}_{4}^{+}$), a form of nitrogen that can be assimilated into organic molecules by plants since plants and other eukaryotes are not able to directly access atmospheric N_2_ (Schwember et al., 2019).

Nitrogen is one of the three primary macronutrients (along with phosphorus and potassium) required for plant growth, and agriculture relies on sustained nitrogen provision to maintain continued productivity. There are many legume species that are used in agriculture, and symbiotic BNF by legumes plays a major role in provision of nitrogen in global agriculture, amounting to 33–46 Tg nitrogen per year for the combination of crop and pasture/fodder legumes (Herridge et al., 2008). Nitrogen is also a widespread limiting nutrient in nonagricultural terrestrial systems, and wild legume species are commonly found in nitrogen‐poor soils, especially in the early parts of disturbance–succession cycles (Gei et al., 2018; Vitousek et al., 2013).

Symbiotic N_2_‐fixing rhizobia are housed in root nodules, where the bacterial cells provide NH_3_/${\text{NH}}_{4}^{+}$ to the plant while the plant provides reduced carbon (ultimately from photosynthesis) to the bacteria (Schwember et al., 2019). This is a tight symbiotic association, and the nodule provides a microaerobic environment for the process, since nitrogenase, the enzyme involved in N_2_ fixation is highly oxygen‐sensitive. However, due to the carbon costs of producing and maintaining root nodules and the rhizobia, and the high ATP requirements of the nitrogenase reaction, legume symbiosis‐derived nitrogen is more bioenergetically expensive than uptake of other easily assimilated forms of nitrogen from the soil (Minchin & Witty, 2005). Biologically available soil nitrogen sources for plants include nitrate (${\text{NO}}_{3}^{-}$ often the most abundant nitrogen source in aerobic temperate soils), ammonium (${\text{NH}}_{4}^{+}$), and amino acids (Miller & Cramer, 2005). Both nitrate and ammonium decrease nodulation in legumes (Murray et al., 2017), as do the amino acid glutamine and the commonly used nitrogen fertilizer urea (Yamashita et al., 2019).

#### 1.1.3 The nitrogen addition experiment—methodology

Students were provided with an overview of the relevant literature and kit materials in the accompanying laboratory manual before they were guided to form an individual hypothesis. Students were then thoroughly guided through the experimental design with pre‐recorded videos, live virtual sessions, and written resources detailing best practices. We also hosted a Twitch Channel during this time for students to view the experiment being performed live on‐campus.

**FIGURE 2:**
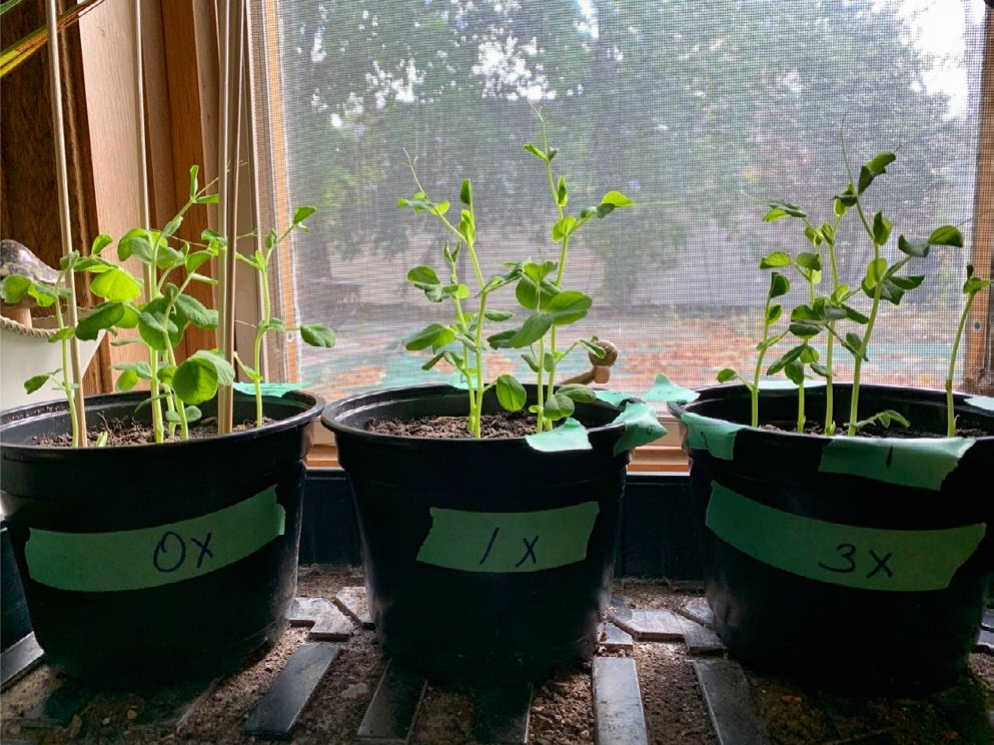
A student at‐home laboratory set up from Fall 2020. Little Marvel seedlings photographed 14 days post‐shoot emergence from soil. Photo: Laura J. Schnell

To study the effect of nitrogen fertilizer on *P*. *sativum* growth and Rhizobia root nodulation, students pregerminated and sowed 6 seeds per treatment condition, including a 0X (control), a 1X treatment, and a 3X treatment (Figure 2). The experimental treatments commenced 7 days following pea shoot emergence. Plants were treated once per week for three consecutive weeks. Students collected measurements three times per week using a data collection template (Simpson & Davis, 2021) and were asked to record all observations in an electronic laboratory notebook. Growth conditions were recorded, including the photoperiod, average temperature, relative humidity (if available), soil type and provenance, soil pH (if available), and frequency of watering. Students measured total plant height from the stem base at the top of the soil to the tip of apical meristem, counted the number of internodes, and made qualitative observations on overall plant color and health. Students harvested plants twenty‐eight days postemergence and were asked to measure fresh weight using a kitchen scale if possible. Students qualitatively assessed root nodulation using the Saskatchewan Pulse Growers Field Assessment Guide, available on GitHub (Simpson & Davis, 2021).

#### 1.1.4 Data analysis using R

Experimental results were collated from the cohort of participating students, who then analyzed the combined data set using R, an open‐source statistical software package that is used to manage, analyze, and visualize data (R Core Team, 2021). Prior to performing data analysis on cohort data, students practiced data management and analyses in RStudio Cloud (Rstudio Team, 2020) on cohort data collected in previous years from the alternative experiment (see below). To avoid software installation and minimize support effort, students were asked to create a free, individual account on rstudio.cloud. Using RStudio Cloud has the added benefits that students can easily share their projects or scripts with instructors if they encounter errors, and they do not require access to a computer on which they can install software as RStudio Cloud is accessed via a web browser. Using this system, instructors could easily replicate errors and help solve the issues effectively and efficiently. Students were guided through data wrangling (organizing, cleaning, reshaping), analysis (statistical modeling and hypothesis testing), and visualization using video screencasts and a companion HTML document built using RMarkdown (Allaire et al., 2021), which detailed all the steps the students were required to take. A rendered version of the RMarkdown document is served at https://simpson‐lab.github.io/plant‐physiology‐lab/, while the necessary data and code to recreate the analysis exercises are available on GitHub (Simpson & Davis, 2021).

#### 1.1.5 Assessments

**FIGURE 3:**
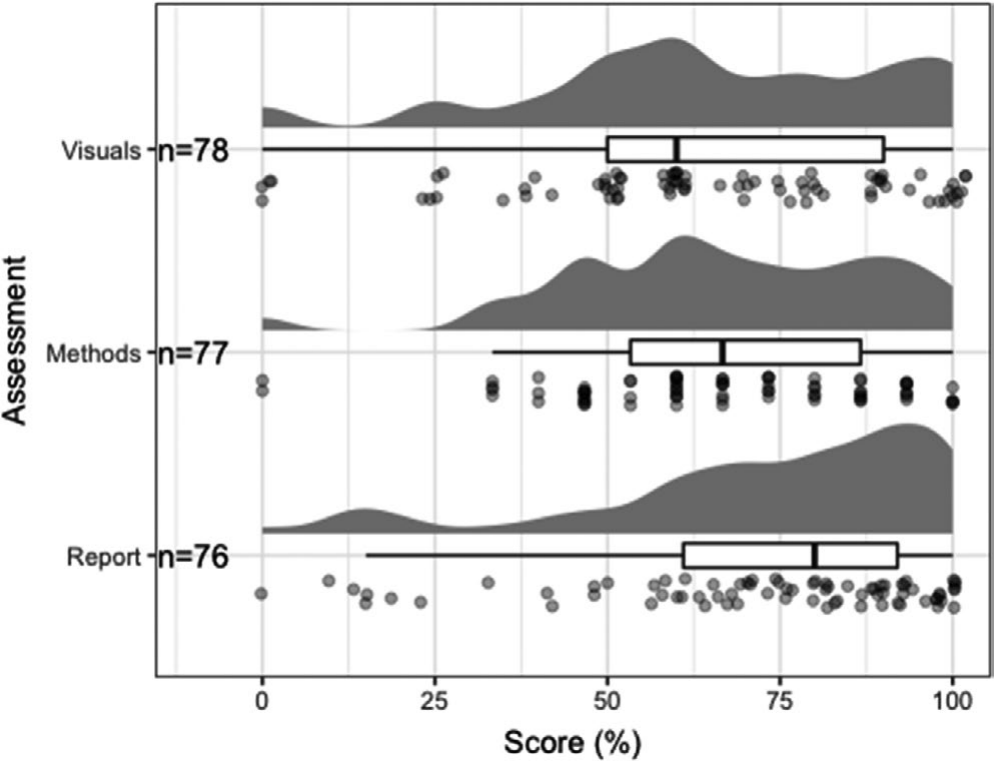
Distribution of student scores (%) in three assessments directly applying the quantitative reasoning bioskill (Clemmons et al., 2020) performed by students over the course of the semester (September–December 2020). The “Visuals” and “Methods” assessment were completed early in the semester (end of September and early October, respectively) and the “Report” was completed in December

The overarching learning outcome of this at‐home laboratory was to compose a research paper, effectively communicating findings to a general biology audience. The final assessment in this laboratory course was a complete research paper including the following sections: Introduction, Methods, Results and Discussion, and Conclusion. Students practiced iterative writing with instructor feedback for each of these sections over the course of the semester in a similar order as done when writing a scientific manuscript (Results & Discussion alongside Methods, ending with Conclusion and Introduction). Early in the semester, after the students began the at‐home experiment, they were guided through data analysis in R using a practice data set (data collected in a previous year). Following this learning exercise, students submitted a Results section (“Visuals” assessment) and were allowed to resubmit this assessment following a round of feedback. To aid students constructing a Methods section, we used an assessment where students were provided with a poorly written Methods section and asked to critique it. Students submitted their Introduction section for peer review and then re‐submitted the updated Introduction (following peer‐review) for instructor feedback. Grading rubrics for all assessments were provided to the class at the start of the semester. Distribution of scores achieved in the assessments described above is shown in Figure 3. A study specifically testing the learning gains in this laboratory model is currently ongoing.

#### 1.1.6 The alternative experiment

To ensure equitable opportunities for achieving learning outcomes, students that were unable to complete the at‐home laboratory were provided with an overview (background information) of an alternative experiment studying the effect of plant hormone gibberellic acid on the development pattern of wild‐type and dwarf pea cultivars in the accompanying laboratory manual (Simpson & Davis, 2021).

Gibberellic acids (GAs), also called gibberellins, are a well‐characterized class of plant hormones (reviewed in Schwechheimer, 2008). Many plant developmental processes are affected by GAs, including the regulation of stem elongation. Mutations in GA metabolism may cause plants to either be shorter and stockier (commonly called dwarf plants) than wild‐type plants, or to be taller and spindlier. Two general classes of GA dwarf mutants are well known: mutants that are deficient in endogenous GA synthesis, and GA‐insensitive mutants that are do not respond to GA (Hedden, 2003). In this experiment, students were provided with an experimental design that included wild‐type and dwarf pea plants, and one of their tasks was to identify the basis of the dwarf phenotype (GA biosynthesis versus GA response).

If the alternative experiment was chosen by students, they were also guided to form a hypothesis based on the background information provided. Following hypothesis generation, students were provided with detailed experimental design, time‐lapse video of pea plant growth (wild‐type and dwarf mutant, +/‐ gibberellic acid treatment) and data including plant height, number of internodes and fresh weight at end of experiment. Students were also guided through the analysis of this data in rstudio.cloud through video screencasts and a companion HTML document built using RMarkdown (Allaire et al., 2021). A rendered version of this RMarkdown document is hosted at https://simpson‐lab.github.io/plant‐physiology‐lab/, while the necessary data and code to recreate the analysis exercises are available on GitHub (Simpson & Davis, 2021). Students were allowed to choose the alternative experiment if they were unable to complete the nitrogen addition experiment for any reason. In some cases, students subsequently opted for the alternative experiment due to the death of most of their experimental plants during the nitrogen addition experiment.

## 2 AT‐HOME LABORATORIES: BENEFITS AND LIMITATIONS

The at‐home laboratory presented in this report allows students to achieve two core competencies, recently termed “bioskills”: (1) process of science and (2) quantitative reasoning (American Association for the Advancement of Science, 2011; Clemmons et al., 2020) that are essential in most prospective STEM career paths. In most biology curricula, bioskills such as generating a hypothesis (question formulation), gathering own data (study design), analyzing their data, and drawing conclusions (data interpretation and evaluation) by doing research is generally reserved for upper division courses (Davis et al., 2020). This at‐home laboratory provided sophomores, in a lower division course, an opportunity to experience authentic research by being guided through course learning outcomes that constitute the process of science bioskill (Clemmons et al., 2020), with a focus on effective written communication of results. To effectively communicate their findings, students must use their current knowledge and think critically to synthesize and apply knowledge acquired through scientific experimentation. In addition to practicing information literacy through the interpretation and summary of primary literature, students also practice effective writing through the giving and receiving of feedback in the process of peer review over the course of the semester.

Effective communication of experimental results depends on effective data management and analysis (quantitative reasoning bioskill). This requires biologists to be able to appropriately format data, perform data quality control checks, identify data input errors, explore data to identify appropriate statistical tests, and produce graphical representations of experimental results (Auker & Barthelmess, 2020). Additionally, students who analyze their own data are more invested and engaged in their inquiry (Cooper et al., 2020). Unfortunately, development of some of these skills is often overlooked in undergraduate curricula due to a lack of available time to focus on such skills in a semester‐long course. In undergraduate teaching, instructors often tend to focus on performing selected statistical tests and producing graphical representations of the data rather than data management and exploratory analysis (Auker & Barthelmess, 2020).

We used the highly flexible and powerful open‐source data analysis software R to introduce students to basic data management and exploratory analyses in this URE. Without prior programming experience, learning R can seem intimidating to both instructors and students alike. This transition can be made less daunting for students using practice data sets to guide them through the data management and exploratory analyses early in the semester. Based on anecdotal evidence from our at‐home laboratory, performing data analysis on practice sets allowed students to feel less overwhelmed when analyzing their own data at the end of the semester. R is increasingly being used in undergraduate curricula (e.g., Auker & Barthelmess, 2020), including in biology, as a cost‐effective and appropriate tool for teaching modern applications of statistics to scientific problems. The use of R was further motivated by a desire to avoid giving students the impression that data wrangling, exploration, and statistical analysis is a point‐and‐click process involving the following of a flow chart to identify the “correct” test to apply in a given situation. Additionally, we discussed the importance of reproducible research and of maintaining a script documenting the steps in the data analysis. We used rstudio.cloud, a free, cloud‐based computing option for students to use from home. This helped solve the issues of students requiring access to a computer upon which they can install their own software and the need for local IT staff to support remote access to on‐campus computing resources. For instructors with little or no R knowledge who are interested in implementing data analysis using R, we suggest collaborating with a statistician at your institution, as was done in this project. We further suggest incorporating data management and analysis using R in courses across biology curricula in partnership with the Statistics Department at your institution, giving students multiple opportunities to practice using R and analyze multiple types of biological data. The data wrangling and statistical methods used in our exercise could be reproduced in other statistical software, although additional steps may be required to ensure students are able to access the required software and have access to suitable support options that provide timely response to queries/errors, and which scale to the number of students in a course.

At‐home UREs bridge the practical and applied skills gap created by the pivot to remote teaching and learning during the COVID‐19 pandemic. Recent educator experiences suggest that open‐ended research projects foster better engagement as at‐home laboratories as opposed to short, strictly directed (“cookbook” type) experiments (Fox et al., 2020). Research‐based at‐home laboratories further encourage independent problem‐solving skills that give students an increased sense of control and confidence (Andrews et al., 2020). At‐home laboratories have the added benefit that the activities or experiments can be completed at one's own pace, or re‐done if necessary. At‐home laboratories may therefore serve to reduce stress and anxiety in students. At‐home UREs also increase student access to authentic, hands‐on research experience, especially at large institutions with class sizes of 75+ students, where students may not always have the opportunity, or the ability to work in a research laboratory due to limited number of such opportunities (Brownell & Kloser, 2015; Cooper et al., 2021; Ward et al., 2014). In these instances, at‐home laboratories can also provide opportunities for authentic research experiences early in students’ undergraduate careers—an intervention that has been demonstrated to increase persistence in STEM fields (Graham et al., 2013; Hanauer et al., 2016; Rodenbusch et al., 2016).

Despite the many ways at‐home UREs can provide increased opportunity for equitable learning, they also come with limitations. At‐home laboratory experiences must be carefully designed to be equitable and inclusive in terms of (1) suitability for a nonlaboratory setting (i.e., at‐home) and (2) allowing all students the opportunity to achieve associated learning goals and outcomes. In addition, equity should be considered not only in terms of resource accessibility and socioeconomic inequalities, but also in terms of student diversity in learning experiences and educational background. These activities must therefore be designed to reasonably accommodate financial, time, and space limitations for students.

To keep student costs low in our at‐home laboratory design, most of the required supplies are provided to students. Students who chose to perform the at‐home experiment need to source soil for the experiment themselves; this can present a challenge for students if they live in an apartment or do not have access to a yard. At‐home laboratories have the potential to require more time than students would otherwise spend performing a tightly scheduled laboratory with instructor setup and maintained experiment, since at‐home UREs require students to set up and monitor the experimental system for the duration of the experiment. Additionally, limited instructor oversight of the daily experimental process, especially with respect to housekeeping items such as seed germination, watering, and ensuring general health of the experimental plants, may lead to unforeseen issues such as overwatering or mold, ultimately leading to loss of experimental plants. In terms of monitoring experimental plants, students are provided with a guide for selecting soils, a recommended watering schedule, and tips monitoring plant health to ensure plants remain healthy over the duration of the experiment. However, these limitations must be taken into consideration when designing assessments and other laboratory‐associated activities.

An at‐home laboratory provides flexibility in terms of monitoring and data collection and can better accommodate student work schedules and family obligations. Students can perform the data collection at a time that works for them and without the added costs of travel to an on‐campus laboratory, which itself may require data collection outside of scheduled laboratory time. Space constraint is another limitation that can deter students from having a successful at‐home laboratory experience. In our experiment, for example, students need space in front of a light source (window or grow lights if available) to effectively grow the experimental plants. To maintain equity in cases where laboratory design or requirements prohibit students from performing the laboratory, an alternative experiment and assessment was provided. Alternative assessments help maintain flexibility and accessibility to the learning outcomes of this at‐home laboratory, especially in cases where students may lose experimental plants early in the experimental timeline. In 2020, 4% of the students opted to perform the alternative experiment as they were unable to perform the at‐home laboratory, while 12% opted to use data from the alternative experiment in their final report due to death of plants during the at‐home experiment.

## 3 CONCLUSIONS

The pivot to online teaching necessitated by the COVID‐19 pandemic has required instructors implement new strategies to help students achieve learning outcomes, especially those associated with scientific research. Though at‐home laboratories come with unique challenges, such as cost (if kits are not provided to students), time, and space to perform the activities, those designed with these challenges in mind can provide students with authentic hands‐on research experiences. In many cases, at‐home laboratories are an opportunity for increased learning equity through flexibility of time, experiment pace, and place as well as increased control and opportunity for more independent data analysis. In this at‐home laboratory, we focused on developing two of the core competencies recommended in Vision and Change in Undergraduate Biology Education: A Call to Action: (1) the ability to apply the process of science, and (2) the ability to use quantitative reasoning (AAAS, 2011). With respect to both competencies, data management and analysis is an essential skill for biologists. We plan to continue this work to assess the learning gains of this at‐home laboratory, specifically investigating student perspectives on, and interest in, this at‐home laboratory, and if those change following the transition back to on‐campus learning. We aim to identify the factors that students categorize as limitations to their learning through at‐home laboratories such that they can be mitigated in future iterations, and aid in the design of a HyFlex and/or Distance Laboratory Courses. The ultimate goal of this work is to increase the flexibility of learning time and place for our students while providing students equitable and inclusive opportunities to engage in an authentic research experience.

## CONFLICT OF INTEREST

None.

## AUTHOR CONTRIBUTIONS

**Laura J. Schnell:** Methodology (equal); Resources (supporting); Writing – original draft (supporting); Writing – review & editing (equal). **Gavin L. Simpson:** Conceptualization (supporting); Data curation (lead); Methodology (supporting); Resources (equal); Software (lead); Writing – review & editing (equal). **Danae M. Suchan:** Conceptualization (supporting); Methodology (supporting); Resources (supporting); Writing – review & editing (equal). **William Quere:** Methodology (supporting); Resources (supporting); Writing – review & editing (supporting). **Harold G. Weger:** Resources (supporting); Writing – review & editing (equal). **Maria C. Davis:** Conceptualization (lead); Methodology (equal); Resources (lead); Writing – original draft (lead); Writing – review & editing (equal).

## Data Availability

All supplementary materials including data can be found here https://www.doi.org/10.5281/zenodo.5338613.
